# Neuroimaging Correlates of 3 Distinct Physical-Cognitive Phenotypes in Cognitively Normal Older Adults: The Gothenburg H70 Cohort Study

**DOI:** 10.1212/WNL.0000000000210121

**Published:** 2024-12-06

**Authors:** Rui Wang, Anna Marseglia, Johan Skoog, Olof Lindberg, Joana B. Pereira, Sara Shams, Mana Shams, Miia Kivipelto, Therese Rydberg Sterner, Silke Kern, Anna Zettergren, Ingmar Skoog, Eric Westman

**Affiliations:** From the Department of Physical Activity and Health (R.W.), the Swedish School of Sport and Health Sciences, GIH, Stockholm; Division of Clinical Geriatrics (R.W., A.M., O.L., S.S., M.S., M.K., E.W.), Center for Alzheimer Research, Department of Neurobiology, Care Sciences and Society, Karolinska Institute, Solna, Sweden; Wisconsin Alzheimer's Disease Research Center (R.W.), University of Wisconsin School of Medicine and Public Health, Madison; Centre for Ageing and Health (AgeCap) (J.S., O.L., T.R.S., S.K., A.Z., I.S.), Neuropsychiatric Epidemiology Unit, Department of Psychiatry and Neurochemistry, Institute of Neuroscience and Physiology, Sahlgrenska Academy at the University of Gothenburg, Mölndal; Department of Psychology (J.S.), University of Gothenburg, Göteborg; Neuro Division (J.B.P.), Department of Clinical Neurosciences, Karolinska Institute, Stockholm; FINGERS Brain Health Institute (M.K.), Stockholm; Medical Unit Aging (M.K.), Karolinska University Hospital, Solna, Sweden; Ageing Epidemiology (AGE) Research Unit (M.K.), School of Public Health, Imperial College London, Medical School Building, St Mary's Hospital, United Kingdom; Institute of Public Health and Clinical Nutrition and Institute of Clinical Medicine (M.K.), Neurology, University of Eastern Finland, Kuopio; Aging Research Center (T.R.S.), Department of Neurobiology, Care Sciences and Society, Karolinska Institutet and Stockholm University; and Department of Psychiatry Cognition and Old Age Psychiatry (I.S.), Sahlgrenska University Hospital, Region Västra Götaland, Mölndal, Sweden.

## Abstract

**Background and Objectives:**

Individuals aged 70 and older frequently experience an increased risk of deficits in both physical and cognitive functions. However, the natural progression and interrelationship of these deficits, as well as their neurologic correlates, remain unclear. We aimed to classify the data-driven physical-cognitive phenotypes and then investigate their associations with neuroimaging markers.

**Methods:**

This cross-sectional study included 70-year-old participants from the Gothenburg H70 Birth Cohort (2014–2016). Based on physical performance (grip strength, balance, walking speed, and chair stand) and cognitive measures (episodic memory, perceptual speed, executive function, verbal fluency, and visuospatial abilities), we applied latent class analysis to identify physical-cognitive phenotypes. Based on the brain MRI measurements, 3 groups of neuroimaging markers were involved—neurodegeneration, cerebral small vessel disease (cSVD), and microstructural white matter (WM) integrity. We performed multinomial logistic regressions to examine the differences between the physical-cognitive phenotypes.

**Results:**

In total, 1,140 participants (female: 53.3%) without dementia and disability were included in the study, with 721 (female: 52.2%) undergoing MRI scans. Three physical-cognitive phenotypes were identified: an “optimal” group characterized by high performance in both physical and cognitive functions, an “intermediate” group showing a slight reduction in both domains, and a “physical deficit” group marked by a significant reduction in physical performance. Compared with the optimal group, the other 2 groups were more likely to present with vascular risk factors. The physical deficit group had higher odds of experiencing depression compared with the intermediate group (adjusted odds ratio [aOR] 2.9, 95% CI 1.4–5.9). Compared with the optimal group, the odds of presenting all 3 severe neuroimaging markers were higher in both the intermediate (aOR 3.4, 95% CI 1.5–7.9) and physical deficit (aOR 10.3, 95% CI 2.4–45.0) groups.

**Discussion:**

This study highlights the variability in physical and cognitive performance among older adults and suggests that neuroimaging markers of neurodegeneration, cSVD, and microstructural WM integrity may account for these variations. Our findings indicate the potential for developing group-based strategies to prevent and manage age-related functional decline. Further research with larger sample sizes is needed to deepen our understanding of physical-cognitive decline patterns.

## Introduction

The term “successful aging” has been used in the gerontological field to refer to the maintenance of optimal physical and cognitive functioning in older adults for as long as possible.^1^ Limitations in these 2 domains, in turn, can predict age-related neurodegenerative diseases, such as dementia.^2,3^ Research indicates that individuals older than 70 years frequently experience slowed physical function and cognitive impairment.^4,5^ Yet, no study has thoroughly examined the heterogeneity and interrelation of these 2 functions in response to aging, particularly in older adults aged 70.^6^

Previous studies have identified the co-occurrence of physical and cognitive decline using predefined cut-off values or clinical diagnoses, such as a walking speed of less than 0.4 m/s and cognitive impairment diagnosis.^7^ This approach may not fully capture the complex interplay between physical and cognitive deficits that occur during the aging process. It remains uncertain which deficit occurs first and how the heterogeneity in these 2 functions presents among older adults. Few studies have used data-driven approaches to classify older adults into distinct groups based on their physical and cognitive function.^6,8^ However, further investigation is needed to validate these physical-cognitive phenotypes using a broader range of measurements.

Neurologic pathologies, such as cerebral small vessel disease (cSVD), neurodegenerative brain changes, and loss of microstructural white matter (WM) integrity, occur in cognitively normal older individuals. These pathologies contribute not only to cognitive impairment and Alzheimer disease but also to limitations in physical function.^9-13^ These age-related neurologic pathologies may represent different underlying mechanisms that explain the distinct physical-cognitive phenotypes observed in aging. Nevertheless, to the best of our knowledge, evidence on the neuroimaging correlates of the classified physical-cognitive phenotypes in older adults is scarce.

Using a population of 70-year-old adults, the current study aims to identify distinct physical-cognitive phenotypes and characterize these phenotypes by linking them to structural neuroimaging markers of cSVD, neurodegeneration, and WM microstructural alterations.

## Methods

### Study Participants and Data Collection

This study involved study participants from the population-based Gothenburg H70 Birth Cohort Study.^14^ Briefly, through the Swedish Tax Agency population register, we invited older adults who were born in 1944 and living in Gothenburg (private households or residential care) to participate in the baseline examination (January 2014–December 2016). Specifically, we invited 1,667 septuagenarians, out of 1,839 initially eligible participants (172 declined or died before the examination), to participate in the baseline examination. In total, 1,203 participants underwent baseline examination (response rate, 72.2%), and 788 of them who were without contraindications (e.g., claustrophobia and metallic implants) underwent the structural brain MRI examination. This cross-sectional study included 1,140 participants at baseline, after excluding 30 with dementia diagnosed, 11 Barthel Index of Activities of Daily Living <60,^15^ 15 with Mini-Mental State Examination (MMSE) score ≤24,^16^ and 7 with incomplete information on cognitive tests. Among the 1,140 older individuals, 721 underwent a brain MRI examination (eFigure 1).

Through semistructured interviews and clinical examinations performed by research nurses and physicians, we obtained data on social demographic factors (e.g., sex and education), height, weight, cardiovascular risk characteristics (e.g., blood pressure, smoking status), medication use, somatic and psychiatric conditions, and markers of peripheral blood samples. We obtained the medical history of participants by linking them to the Swedish National Patient Register. Information on in vivo biomarkers of brain pathology was collected in subsamples using CSF sampling and structural brain MRI examinations.^14^

### Physical Performance Assessment and Neuropsychological Assessment

Physical performance tests included the walking speed test, grip strength test, timed chair-stand test, and 1-leg static balance test.^14,17-20^ We recorded walking time using a self-selected gait speed test over a 30-m distance indoors, starting from a standing position.^17^ Grip strength was measured using a Martin Vigorimeter, with the shoulder in a neutral position, and the average value of both hands was recorded.^20^ We assessed the total time spent on 5 chair-stand tests as another measure of physical performance.^18^ Balance was evaluated by estimating the time spent on 1-leg static balance tests.^19^ To ensure consistency, we reversed the scores for walking time over 30 m and the total time spent on the chair-stand test, so that higher values indicate better performance. Subsequently, we standardized each physical performance measure into *z*-scores, which were applied to our analyses.

Participants performed a neuropsychological test battery that comprised 10 tests.^14^ Five subdomains of cognitive performances were evaluated by the neuropsychological test battery, including executive function (Digit Span Backward and Figure Logic),^21^ episodic memory (derived from the Memory in Reality–free recall and 12-object delayed recall, and Thurstone's picture memory),^22^ attention or perceptual speed (Figure Identification-Psif and Digit Span Forward),^21^ verbal fluency (phonemic fluency derived from the Controlled Oral Word Association-FAS and semantic fluency derived from “animals” task),^23^ and visuospatial abilities (Koh's block test).^24^ We standardized the original test score in each sub-domain into *z*-scores and applied them in the analyses.

### Sociodemographic Factors, *APOE* ε4 Status, Cardiovascular Health, and Depression

Education was recorded as the maximum years of receiving formal education. *APOE* single-nucleotide polymorphism was genotyped using DNA extracted from the blood samples.^14^ Participants were categorized as *APOE* ε4 carriers or noncarriers. Smoking status was categorized into never, former (age of quitting 50 years or younger, age of quitting 50–70 years), or current smoking. According to the National Institute on Alcohol Abuse and Alcoholism guidelines, alcohol consumption was categorized as recommended (<100 g of alcohol per week) vs at-risk consumption (≥100 g per week).^25^ A short version of the International Physical Activity Questionnaire was applied to quantify physical activity levels.^26^ Weekly physical activity amount was quantified as transformed metabolic equivalent (MET) minutes per week. According to the physical activity guideline, our study participants were categorized into 3 groups: low (<600 MET), moderate (600–2,999.9 MET), or high (≥3,000 MET) levels. We calculated body mass index (BMI) using the ratio of weight to squared height (kg/m^2^) and categorized normal weight (BMI <25 kg/m^2^), overweight (BMI 25–29.9 kg/m^2^), and obesity (BMI ≥30 kg/m^2^).^27^ Hypertension was defined by blood pressure (systolic blood pressure ≥140 mm Hg/diastolic blood pressure ≥90) or self-reported antihypertensive medication use.^28^ Diabetes was defined according to self-reported medical history, self-reported medication use of glucose-lowering treatments, or fasting/nonfasting blood glucose ≥7.0/11.1 mmol/L.^29^ Cholesterol status was categorized as high (>6.18 mmol/L), intermediate (5.172–6.18 mmol/L), and optimal (<5.172 mmol/L) levels.^30^ Heart diseases were identified based on the presence of myocardial infarction, angina pectoris, heart failure, and atrial fibrillation. Depression was diagnosed according to the *Diagnostic and Statistical Manual of Mental Disorders, fourth or fifth edition* revised criteria.^31^

### Neuroimaging Acquisition Protocol and Markers

MRI was performed on a 3.0T Philips Achieva system (Philips Medical Systems) following a standard protocol. Detailed information on the parameters in the MRI protocol and the neuroimaging processing software and server are summarized in eTable 1.

#### Markers of cSVD

WM lesion (WML) volume was automatically segmented using the Lesion Segmentation Toolbox (LST 2.0.15) in the SPM software.^32^ The total WML volume has been further adjusted for total intracranial volume (TIV).^33^ Besides the automatic segmentation, visual assessments on vascular brain pathologies were performed by an experienced neuroradiologist.^34^ We obtained Fazekas score using FLAIR images, and each participant's deep WML load was visually graded as follows: 0 = absence of WML, 1 = punctate WML, 2 = early confluent WML, and 3 = WML in large confluent areas. Based on the FLAIR images, the number of lacunes (lacunar infarcts in size 3–15 mm^3^) were visually rated.^34^ Enlarged perivascular spaces (EPVSs) were rated according to Mac Lullich's rating scale.^34^ The number of EPVS was recorded as 0 (none EPVS), 1 (1–10 EPVS), 2 (11–20 EPVS), 3 (21–40 EPVS), and 4 (EPVS ≥40). The EPVS scales were determined for the basal ganglia and centrum semiovale regions. To quantify the severity of cSVD, a cSVD score was calculated. The score was determined by considering the number of (1) severe WML (Fazekas score = 3 or falling within the top tertile of WML volume), (2) the presence of severe EPVS in either region (scale ≥3), and (3) the presence of 1 or more lacunes.^35^

#### Markers of Neurodegeneration

We conducted cortical reconstruction and volumetric segmentation on the T1 images through the Freesurfer image analysis pipeline.^34^ Cortical thickness was measured as the distance from the gray/WM boundary to the corresponding pial surface. Mean cortical thickness across 34 mantles of interest was used in this study. In addition to measuring cortical thickness, we involved the average volume of the hippocampus from both hemispheres, gray matter (GM) volume, and total ventricular volume as the individual markers of neurodegeneration. To account for individual differences in brain size, these markers were adjusted for TIV.^33^ A neurodegenerative score was computed to represent the severity of neurodegeneration, by considering the number of severe reductions in cortical thickness (the bottom tertile of average cortical thickness), severe reductions in GM (the bottom tertile of total GM volume), severe reductions in hippocampus (the bottom tertile of hippocampal volume), and severe enlargement of ventricles (the top tertile of ventricular volume).^36^

#### Markers of Microstructural WM Integrity

Using the FMRIB's Diffusion Toolbox from FSL, diffusion-weighted images were analyzed to obtain the measurements of microstructural WM integrity.^34^ We computed the fractional anisotropy (FA) and mean diffusivity (MD) maps for each voxel to obtain information on microstructural WM integrity. Using the tract-based spatial statistics tool, the FA and MD maps were transformed into Montreal Neurological Institute space, and FA/MD values across 26 regions of interest were extracted to calculate the average FA and MD values. A lower value in FA and a higher value in MD indicate a reduced microstructural WM integrity.^37^ To evaluate the severity of reduced microstructural WM integrity, we developed a WM microstructural score consisting of 2 values: 0 or 1. A score of 1 was assigned based on whether the FA value fell within the bottom tertile group or the MD value fell within the top tertile group.

### Statistical Analysis

The characteristics of the analytical sample, non-MRI sample, and MRI subsample were presented with means (SD), median (interquartile range), or frequencies (n, %). The differences in characteristics between non-MRI and MRI samples were compared using *t* tests for continuous variables or chi-square tests for categorical variables. For continuous variables that did not follow a normal distribution, the Wilcoxon rank-sum test was applied.

Latent Class Analysis (LCA) with continuous items was conducted to identify the physical-cognitive phenotypes. LCA is a person-centered approach that tries to discover the minimum number of latent classes describing the associations among a set of observed variables.^38^ To find the best class solutions, we compared the Akaike Information Criteria (AIC), Bayesian Information Criteria (BIC), reduction of AIC/BIC, class size, entropy, and average latent class posterior probability from different models. The final LCA model was determined by integrating these parameters. Based on the observed variables (4 physical performance variables and 5 tests of subcognitive domains), LCA identified unobserved groups among our participants who were of the same age.

In the baseline sample (n = 1,140), multinomial logistic regression was performed to compare the differences in *APOE* ε4 status, vascular risk factors, heart diseases, and depression between the distinct physical-cognitive phenotypes that were identified by LCA. Results were presented as the odds ratio (OR) with 95% CIs, or average marginal effects.

To investigate the relationships between neuroimaging markers and the distinct physical-cognitive phenotypes, we assessed the associations of individual neuroimaging markers, the cSVD severity score, neurodegenerative severity score, and WM microstructural score with the respective physical-cognitive phenotypes in the MRI sample (n = 721). We explored the connections between coexisting neuroimaging markers and physical-cognitive phenotype groups. To visualize the distribution of physical-cognitive phenotype groups based on the absence, individual presentation, and coexistence of the neuroimaging markers, we employed a Sankey diagram. Multinomial logistic regression was performed to further examine the likelihood of coexisting neuroimaging markers in different physical-cognitive phenotypes.

All analyses and graphics were performed using software STATA version 17.0 (Stata Corp., College Station, TX) and the statistical software package R version 4.0.5 (R Development Core Team, Vienna, Austria).

### Standard Protocol Approvals, Registrations, and Patient Consents

The H70 study was approved by the Regional Ethical Review Board in Gothenburg and the Radiation Protection Committee (approval numbers: 869-13, T076-14, T166-14, 976-13, 127-14, T936-15, 006-14, T703-14, T201-17, T915-14, 959-15, and T139-15). Written informed consent was obtained from all participants in this study.

### Data Availability

Anonymized data can be obtained by reasonable request from any qualified investigator.

## Results

### Characteristics of the Study Sample

In the total analytical sample (n = 1,140), 53.33% were women, and the median education level was 13 years (Table 1). Seven hundred twenty-one individuals had undergone a brain MRI examination. No statistical difference was observed between the non-MRI and MRI samples regarding demographic factors, *APOE* ε4 status, smoking status, alcohol consumption, physical activity, BMI, total cholesterol level, hypertension, diabetes, depression, verbal fluency, balance, and chair stand. However, compared with the non-MRI sample, the MRI sample showed fewer heart diseases and better performance in episodic memory, executive function, visuospatial test, grip strength, and walking speed.

**Table 1:** Baseline Characteristics of Study Participants Between MRI and Non-MRI Samples

	Total analytical sample (n = 1,140)	MRI scan		*p* Value
		No (n = 419)	Yes (n = 721)	
Age, y, median (IQR)	70.55 (70.40–70.79)	70.55 (70.41–70.80)	70.54 (70.39–70.72)	0.11
Female, n (%)	608 (53.33)	232 (55.37)	376 (52.15)	0.29
Education year,^a^ median (IQR)	13 (10–16)	12 (10–16)	13 (10–16)	0.08
*APOE* ε4 carriers,^a^ n (%)	356 (32.45)	126 (31.74)	230 (32.86)	0.70
Smoking status,^a^ n (%)				
Never	429 (37.73)	147 (35.25)	282 (39.17)	
Ever	604 (53.12)	221 (53.00)	383 (53.19)	
Current	104 (9.15)	49 (11.75)	55 (7.64)	0.05
Alcohol consumption,^a^ n (%)				
>100 g per week	376 (33.07)	131 (31.49)	245 (33.98)	0.39
Physical activity level,^a^ n (%)				
Low (<600 MET/week)	87 (9.41)	30 (9.74)	57 (9.24)	
Moderate (600–2,999.9 MET/week)	477 (51.57)	163 (52.92)	314 (50.89)	
High (≥3,000 MET/week)	361 (39.03)	115 (37.34)	246 (39.87)	0.76
BMI, kg/m^2^, mean (SD)	26.06 (4.48)	26.25 (4.85)	25.95	0.27
Total cholesterol, mmol/L,^a^ mean (SD)	5.50 (1.17)	5.43 (1.09)	5.53 (1.22)	0.92
Hypertension, n (%)	633 (55.53)	235 (56.09)	398 (55.20)	0.77
Diabetes, n (%)	177 (15.53)	70 (16.71)	107 (14.84)	0.40
Heart diseases,^a^ n (%)	97 (8.52)	46 (11.00)	51 (7.07)	0.02
Depression,^a^ n(%)	98 (8.62)	41 (9.86)	57 (7.91)	0.26
Composite score in the neuropsychological test battery				
Episodic memory,^a^ mean (SD)	0.004 (0.71)	−0.059 (0.67)	0.040 (0.74)	0.02
Perceptual speed,^a^ mean (SD)	0.019 (0.79)	−0.037 (0.81)	0.050 (0.77)	0.07
Executive function,^a^ mean (SD)	0.008 (0.79)	−0.062 (0.83)	0.048 (0.77)	0.03
Verbal fluency,^a^ mean (SD)	−0.008 (0.87)	−0.037 (0.92)	0.009 (0.84)	0.39
Visuospatial abilities,^a^ mean (SD)	0.013 (0.87)	−0.111 (1.02)	0.079 (0.97)	0.00
Physical performance tests				
Grip Strength, kPa,^a^ median (IQR)	73.33 (63.83–83.33)	71.17 (62–82.67)	73.67 (64.33–83.33)	0.03
Balance, s,^a^ median (IQR)	22.67 (12.17–30.00)	21.17 (11.75–30)	23.67 (12.50–30)	0.11
Walking speed, s/m,^a^ median (IQR)	0.65 (0.60–0.72)	0.65 (0.61–0.73)	0.65 (0.60–0.70)	0.01
Chair stand, s,^a^ median (IQR)	12 (10–15)	12 (10–15)	12 (10–14)	0.05

Abbreviations: BMI = body mass index; IQR = interquartile range; MET = metabolic equivalent.

a There was 1 missing value for education year (non-MRI sample), 43 for *APOE* ε4 status (22 in non-MRI and 21 in MRI samples), 3 for smoking status (2 in non-MRI and 1 in MRI samples), 3 for alcohol consumption (non-MRI sample), 215 for physical activity status (111 in non-MRI and 104 in MRI samples), 3 for BMI (2 in non-MRI and 1 in MRI samples), 9 for total cholesterol (5 in non-MRI and 4 in MRI samples), 1 for heart diseases (non-MRI sample), 3 for depression (non-MRI sample), 6 for episodic memory, 19 for perceptual speed, 21 for executive function, 7 for verbal fluency, 68 for visuospatial abilities, 23 for grip strength, 103 for balance, 45 for walking speed, and 60 for chair stand.

### Distinct Physical-Cognitive Decreasing Groups and Their Characteristics

**Figure 1:**
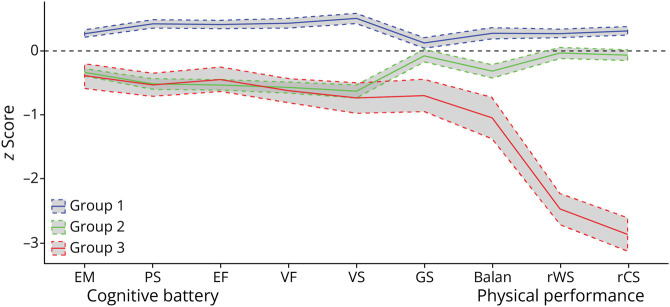
Mean Estimates (95% CI) of Cognitive Battery and Physical Performance From Latent Class Analysis We applied standardized scores for cognitive performance across 5 subdomains, and we standardized grip strength, time spent on balance tests, reversed walking speed (10 m/s), and reversed time spent on chair stand test. Group 1, the optimal group; group 2, the intermediate group; group 3, the physical deficit group. Balan = balance tests; EF = executive function; EM = episodic memory; GS = grip strength; PS = perceptual speed; rCS = reverse chair stand test; rWS = reversed walking speed; VF = verbal fluency; VS = visuospatial abilities.

Using LCA with various class solutions among our study participants, we identified 3 subgroups. eTables 2 and 3 provide the model fit statistics and diagnostic criteria for the various class solutions and correlation matrix of measurements. Figure 1 shows the estimated means with 95% CIs of all observational variables across the 3 sub-groups. There were 622 participants classified in group 1 (54.56%), 458 in group 2 (40.18%), and 60 (5.26%) in group 3. Compared with the other 2 groups, group 1 exhibited the best performance across all cognitive domains and physical functioning (namely, the “optimal” group). Group 2 displayed a gradual reduction in both domains compared with group 1 (“intermediate” group). While no significant difference in cognitive performance was observed between group 2 and group 3, group 3 displayed a notable deterioration in physical performance compared with group 2 (“physical deficit” group). The characteristics of study participants between the 3 physical-cognitive phenotypes were shown in eTable 4, and we did not observe statistical differences between the 3 groups regarding age and sex. The optimal group exhibited a higher education level compared with the other groups, with a median of 14 years of education, whereas the median was 11 years in the intermediate group and 10 years in the physical deficit group.

**Figure 2:**
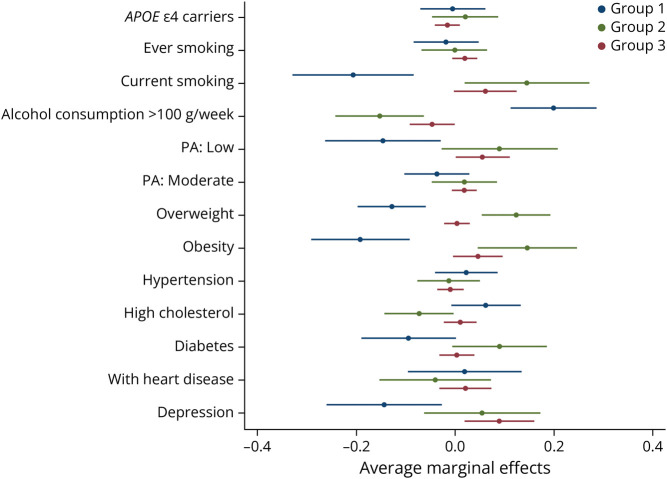
Physical-Cognitive Phenotypes and Their Correlation With *APOE* ε4 Allele, Vascular Risk Factors, Heart Disease, and Depression The average marginal effects were generated after the multinomial logistic regression model, indicating the estimated probability of having the risk factors in each identified group. A higher value of the average marginal effect suggests a higher probability of having the risk factor presented in that group. Group 1, the optimal group; group 2, the intermediate group; group 3, the physical deficit group. PA = physical activity.

The age, sex, and education-adjusted multinomial logistic regression models showed that, compared with the optimal group (group 1), individuals in the other 2 groups were more likely to be smokers (ever and current smokers), low alcohol consumers (≤100 g per week), less physically active, suffering from overweight/obesity, to have a reduced total cholesterol level, more often diabetes, heart diseases, and depression. When all risk factors were included in the same model (Figure 2), the results indicated that current smoking, light or nondrinking, physical inactivity, overweight/obesity, and diabetes were the major factors associated with the intermediate group (group 2) and the physical deficit group (group 3). While vascular characteristics were similar between the intermediate and physical deficit groups, participants in the physical deficit group experienced depression more frequently than those in the intermediate group in the multivariable-adjusted model (OR in the multivariable-adjusted model: 2.91, 95% CI 1.44–5.89).

### The Distinct Physical-Cognitive Functioning Patterns and Their Neuroimaging Correlates

In the subpopulation who underwent MRI scan (n = 721), 417 (57.84%) participants were in the optimal group, 271 (37.59%) participants were in the intermediate group, and 33 (4.58%) participants were in the physical deficit group. When compared with the optimal group, the other 2 groups showed a higher likelihood of having greater WML volume, higher Fazekas deep WML scores, reduced cortical thickness, reduced total GM volume, increased ventricular volume, reduced FA, and increased MD, after adjusting for demographic factors, *APOE* ε4, vascular risk factors, heart diseases, and depression (see eTables 5 and 6). Compared with the intermediate group, the physical deficit group presented more WML volume, reduced cortical thickness, decreased total GM volume, and lower FA values.

When we studied the association between the aggregated scores of the 3 neuroimaging marker groups and the distinct physical-cognitive phenotypes, we found that severe cSVD (cSVD severity score ≥2), significant reduction in neurodegenerative markers (total neurodegenerative score ≥2), and evident decrease in WM microstructure (WM microstructural score = 1) were all more common in the intermediate and physical deficit groups than in the optimal group (Table 2). However, the associations with the cSVD score disappeared when we additionally adjusted for vascular risk factors, heart diseases, and depression. No significant difference in the 3 neuroimaging markers was observed between the intermediate group and the physical deficit group.

**Table 2:** Associations Between Neuroimaging Marker Groups and Distinct Physical-Cognitive Phenotypes

	Model 1^a^ Odds ratio (95% CI)		Model 2^a^ Odds ratio (95% CI)	
	Group 2 (n = 271)^b^ Intermediate group	Group 3 (n = 33)^b^ Physical deficit group	Group 2 (n = 271)^b^ Intermediate group	Group 3 (n = 33)^b^ Physical deficit group
Total cSVD score				
0 (Absence), n = 361	Ref.	Ref.	Ref.	Ref.
1 (Mild), n = 242	0.94 (0.65–1.36)	2.06 (0.89–4.78)	0.91 (0.62–1.34)	1.73 (0.68–4.40)
≥2 (Severe), n = 118	1.74 (1.09–2.76)*	3.05 (1.12–8.25)*	1.51 (0.92–2.45)	1.70 (0.55–5.24)
*p* for linear trend	0.060	0.025	0.219	0.329
Total neurodegenerative score				
0 (Minor decrease), n = 244	Ref.	Ref.	Ref.	Ref.
1 (Mild decrease), n = 201	1.57 (1.03–2.41)*	1.39 (0.43–4.46)	1.56 (1.00–2.43)	1.30 (0.36–4.76)
≥2 (Severe decrease), n = 276	2.39 (1.61–3.55)**	4.49 (1.74–11.60)**	2.36 (1.55–3.59)**	5.28 (1.83–15.24)**
*p* for linear trend	<0.001	<0.001	<0.001	<0.001
Reduced WM microstructural score				
Minor/no decrease, n = 422	Ref.	Ref.	Ref.	Ref.
Severe decrease, n = 299	1.48 (1.06–2.08)*	2.84 (1.33–6.06)**	1.42 (0.99–2.04)	2.74 (1.16–6.46)*
*p* for linear trend	0.022	0.007	0.054	0.021

Abbreviations: cSVD = cerebral small vessel disease; WM = white matter.

a Model 1 was adjusted for sex, education, and *APOE* ε4 status. Model 2 was additionally adjusted for vascular risk factors (smoking status, physical activity levels, obesity, hypertension, diabetes), heart diseases, and depression.

b Reference group is the optimal group.

*0.01 < *p* < 0.05; **p < 0.01.

**Figure 3:**
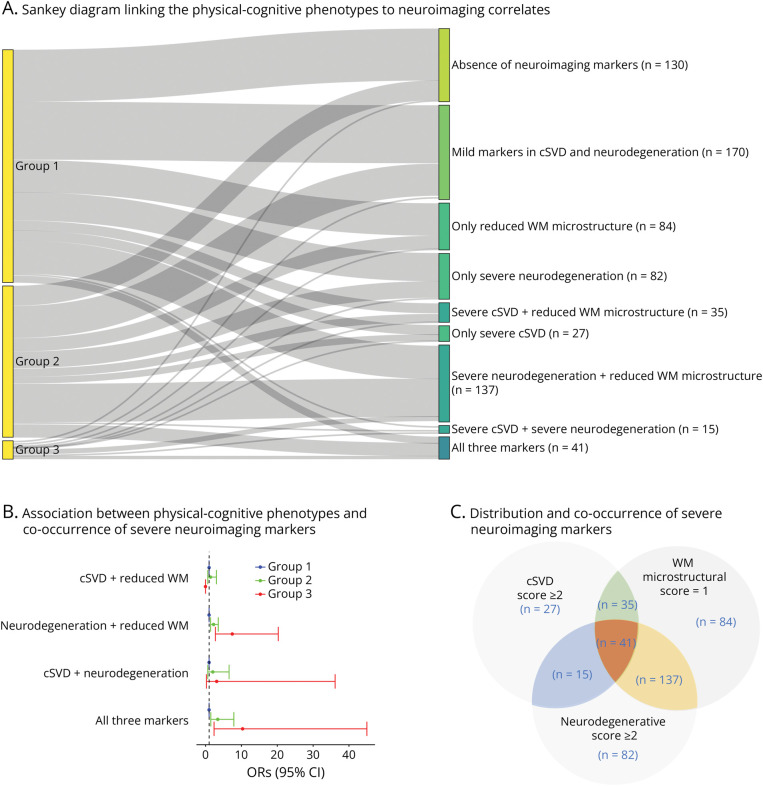
Association Between Physical-Cognitive Phenotypes and Coexisting MRI Markers Group 1, the optimal group; group 2, the intermediate group; group 3, the physical deficit group. (A) Absence of neuroimaging markers: All scores (cSVD, WM microstructural, neurodegenerative) = 0. Mild markers in cSVD & neurodegeneration: WM microstructural score = 0, cSVD and neurodegenerative scores = 1. Only reduced WM microstructure: Only WM microstructural score = 1. Only severe neurodegeneration: Only neurodegenerative score ≥2. Only severe cSVD: Only cSVD score ≥2. Severe cSVD + reduced WM microstructure: cSVD score ≥2 and WM microstructural score = 1. Severe neurodegeneration + reduced WM microstructure: Neurodegenerative score ≥2 and WM microstructural score = 1. Severe cSVD + severe neurodegeneration: Both cSVD and neurodegenerative scores ≥2. All 3 markers: cSVD and neurodegenerative scores ≥2 and WM microstructural score = 1. (B) In this analysis, the reference group combined those labeled “Absence of neuroimaging markers,” “Mild markers in cSVD & neurodegeneration,” and those with only 1 severe neuroimaging marker. This model was adjusted for age, sex, education *APOE* ε4 status, vascular risk factors (smoking status, physical activity levels, obesity, hypertension, diabetes), heart diseases, and depression. See more detailed results in eTable 8. (C) In total, there were 118 participants with a cSVD score ≥2, 275 with a neurodegenerative score ≥2, and 297 with a WM microstructural score = 1. The overlapping circles illustrate the number of participants with co-existing severe neuroimaging markers. The green overlapping area (n = 35) represents those labeled “Severe cSVD + reduced WM microstructure,” the yellow area (n = 137) represents those labeled “Severe neurodegeneration + reduced WM microstructure,” the blue area (n = 15) represents those labeled “Severe cSVD + severe neurodegeneration,” and the orange area represents those labeled “All 3 markers.” eFigure 4 displayed the associations between physical-cognitive phenotypes and co-occurrence of severe neuroimaging markers on log scales. cSVD = cerebral small vessel disease; OR = odds ratio; WM = white matter.

Figure 3 showed associations between the 3 physical-cognitive phenotypes and co-existing severe neuroimaging markers. Figure 3, A and C presented the groups classified by the presence or combination of the 3 neuroimaging scores. Out of the total 721 participants who underwent MRI scans, 300 (41.6%) either had no mild/severe neuroimaging markers or only mild markers related to cSVD and neurodegeneration. Approximately 27% (n = 193) exhibited only 1 severe neuroimaging marker. The co-occurrence of severe neuroimaging markers was observed as follows: 35 participants (4.8%) had both severe cSVD and reduced WM microstructure, 137 participants (19.0%) had severe neurodegeneration combined with reduced WM microstructure, and 15 participants (2.1%) had both severe cSVD and severe neurodegeneration. In addition, 41 participants (5.7%) presented with all 3 severe neuroimaging markers. Figure 3B shows that, compared with the optimal group (group 1), the other 2 groups had a higher likelihood of presenting with coexisting severe neuroimaging marker. In the multivariable-adjusted model, a significant difference between the intermediate group (group 2) and the physical deficit group (group 3) was observed in the coexistence of severe neurodegenerative markers and reduced WM microstructure (OR for group 3 vs group 2 = 3.38, 95% CI 1.25–9.14). Detailed results regarding the physical-cognitive phenotypes and the combination of neuroimaging markers are provided in eTable 7.

### Additional Analyses

eFigure 2 showed the association between the 3 phenotypes and additional vascular risk categories. We conducted analyses excluding individuals with an MMSE score lower than 27, and the 3 classes remained unchanged (see eFigure 3).

## Discussion

The key findings of this study can be summarized in 2 points. First, among older individuals aged 70 years, we identified 3 distinct physical-cognitive phenotypes: an optimal group, an intermediate group, and a physical deficit group. Second, in comparison with the optimal group, the other 2 groups exhibited a higher prevalence of vascular risk factors and demonstrated more severe neuroimaging markers in cSVD, neurodegeneration, or reduced WM microstructure. In contrast to the intermediate group, the physical deficit group was more likely to experience depression and exhibited combined severe markers of neurodegeneration and reduced WM microstructure.

The age-related concurrent decline in physical function and cognition is prevalent in the older population and is highly correlated with an increased risk of dementia as well as the risk of falls. Reduced gait speed has been highlighted as a significant method in assessing dementia risk, and it has been proposed that a dual decline in gait speed and memory may be the best combination to predict future dementia.^39^ Studies thus attempt to disentangle the nature of decline patterns in physical performance and cognition among older individuals through classification analyses, with the most common focus being devoted to joint gait-cognition patterns. In line with our findings, 1 study involving Mexican-American and European American older adults observed 3 distinct gait-cognition decline groups during a 10-year follow-up period—a stable cognition and gait group, a cognitive and gait vulnerability group, and a gait vulnerability group.^6^ Results from the LonGevity study of Ashkenazi Jewish elderly further revealed that gait declined faster than cognition during aging, and the age-related decline rates in both domains were accelerated by poor health conditions.^8^ The longitudinal evidence emphasizes that heterogeneity exists in the joint gait-cognition declines, particularly among cognitively asymptomatic older individuals. However, the impact of the aging process per se on these diversities remains uncertain because most aging cohorts encompass a wide age range. Our study contributes to current evidence by identifying distinct physical-cognitive phenotypes in older adults of the same age, effectively minimizing age-related confounding effects. In addition, we observed that the group with significant reduction in physical performance showed deterioration across all tasks, including grip strength, walking speed, balance, and chair standing.

The pathologic changes leading to joint physical-cognitive decrease or deficit patterns are largely unknown. This study characterizes, for the first time, physical-cognitive phenotypes based on *APOE* ε4 status, health measurements, and neuroimaging markers. We found that the intermediate group and physical deficit group presented more traditional vascular risk factors than the optimal group. This finding ties well with previous studies that have shown an association between vascular risk factors with both physical and cognitive deterioration in older populations.^40,41^ We found that in comparison with the optimal group, the other 2 groups were more likely to be nondrinkers and light drinkers.^41^ Yet further investigations are requested to clarify the link between alcohol consumption and the distinct physical-cognitive decrease pattern. A targeted review has stated that cortical control of gait in aging is bilateral, widespread, and dependent on the integrity of both GM and WM.^42^ A study involving 484 nondemented elderly persons (age range 50–85 years) who all had cSVD indicated that reduced microstructural integrity in both WML and normal-appearing WM was associated with gait disturbances.^43^ Overall, our findings are consistent with the conclusion that the severity of cSVD and neurodegeneration, as well as the reduction in microstructural WM integrity, were quantified differently between the optimal group and the 2 other groups.

Given the observed differences in microstructural WM integrity among the 3 groups, it is evident that changes in WM integrity could have a significant impact on both physical and cognitive decline. Consistent with our findings, previous studies illustrated that loss of integrity in corticostriatal fibers, thalamostriatal fibers, corticospinal fibers (the long descending motor fibers), and frontal–subcortical circuits may contribute largely to the WML-induced functional decline, especially responsible for limiting the lower extremity motor control (e.g., selection, initiation, direction, speed, and duration of movements) and reducing cognitive capacity in executive function.^44-46^ Our findings provide evidence that the coexistence of severe cSVD, neurodegeneration, and reduced microstructural WM integrity—particularly when severe neurodegeneration coincides with reduced WM microstructure—plays a significant role in distinguishing the 3 physical-cognitive phenotypes. These markers may contribute to the deterioration of both cognitive decline and physical performance limitations. Future investigations should focus on examining specific regions related to physical function and cognition in relation to these neuroimaging markers, especially microstructural WM tracts. In addition, both neurodegenerative markers and cSVD markers are linked to reduced WM microstructural integrity (see eTable 8). More evidence is needed to clarify the interrelationships among these 3 neuroimaging markers and their association with physical and cognitive patterns.

The underlying mechanisms of the bidirectional relationship between physical and cognitive performance in the long term have not been fully elucidated. Multiple pathways may link motor and physical performance to cognition, and coexisting damage in various organs and systems may be among the most plausible interpretations. A conceptual model previously proposed suggests that the interaction between physical deficits and cognitive decline becomes more pronounced with advancing age, as cognitive capacities such as attention, coordination, processing, and motor control deteriorate in older adults.^47^ One explanation is that brain alterations occur during the aging process, including reduced vascular brain health, presentation of neurodegenerative pathology, and loss of integrity in brain structural networks. These brain alterations may influence the degree of brain involvement in coordinating cognitive control and age-related decline in motor or physical function, which is known as cognitive-motor interference.^48^ The exact disruption of cerebral networks concerning WML-related physical-cognitive decrease has not been fully understood yet. The frontal basal ganglia–thalamocortical circuit, the limbic area, and the brain stem seemed to be the main vulnerable areas to respond to the vascular lesion and the cognitive-motor interferences.^49^

To the best of our knowledge, this is the first study to investigate distinct physical-cognitive decline patterns among older adults at 70 years. Other strengths include the fact that (1) the study sample was from a systematically selected population; (2) the physical examinations were conducted comprehensively and included performance tests beyond walking speed; (3) a wide range of cognitive tests and physical performances were involved; (4) health conditions and diagnosed diseases (e.g., vascular risk factors, depression, dementia, and heart diseases) were set in accordance with either standard guidelines or established diagnostic criteria; (5) structural brain characteristics were examined thoroughly and covered a comprehensive range of neuroimaging markers in cSVD, neurodegeneration, and microstructural WM integrity; (6) LCA served as a powerful analytical approach for identifying hidden physical-cognitive subgroups within a population of the same age.^50^

Some limitations need to be acknowledged. First, the cross-sectional design of the current study limited us to evaluating the physical-cognitive decline patterns over time, although it is useful for determining preliminary evidence in planning a future investigation. Future longitudinal cohort studies are needed to understand better the temporal patterns of physical and cognitive decline in aging. Second, our study participants were of the same age—70 years—so caution is required when generalizing our findings to other age groups. The physical and cognitive resilience to aging may vary among participants with the same chronological age but different biological age. Third, our analyses have attempted to capture the physical-cognitive phenotypes by targeting specific physical and cognitive tasks, while we are limited to what we have included in our LCA model. Future studies are required to give more attention to other tasks that can catch both cognitive and physical capacity, such as dual-task performance. Fourth, the LCA was conducted on participants who attended the baseline physical examination in the H70 study, with a subset having available neuroimaging markers. Those who underwent MRI scans seemed to have better physical and cognitive performance than those who did not, resulting in a smaller proportion of the physical deficit group in the MRI sample (n = 33, 4.6% vs n = 60, 5.3%). The neuroimaging results, particularly concerning the physical deficit group, need to be further validated in future studies because of the potential for sparse data bias. In addition, although the missing values in the physical performance variables do not seem to affect group identification (eTable 9), future studies may consider handling the missing data in LCA using the full information maximum likelihood method. A deeper understanding of the specific data-driven physical-cognitive phenotypes, along with their potential underlying mechanisms in older individuals without cognitive symptoms, holds significant implications for aging research.

Our findings indicate that different pathologic phases may exist in the interplay between physical and cognitive aging. The coexisting neuroimaging markers in cSVD, neurodegeneration, and reduced WM microstructure seem to be associated with the 3 distinct physical-cognitive phenotypes. The insights from this study could potentially influence future public health intervention strategies for individuals aged 70 years, by customizing methods to address the characteristics of specific phenotypes and groups in diverse contexts. Additional research with larger MRI samples is crucial to confirm the relationships between various neuroimaging markers, such as β-amyloid positivity, and physical-cognitive phenotypes, using a supervised learning approach.

## Appendix Authors

**Table:**

Name	Location	Contribution
Rui Wang	Department of Physical Activity and Health, the Swedish School of Sport and Health Sciences, GIH, Stockholm; Division of Clinical Geriatrics, Center for Alzheimer Research, Department of Neurobiology, Care Sciences and Society, Karolinska Institute, Solna, Sweden; Wisconsin Alzheimer's Disease Research Center, University of Wisconsin School of Medicine and Public Health, Madison	Drafting/revision of the manuscript for content, including medical writing for content; study concept or design; analysis or interpretation of data
Anna Marseglia	Division of Clinical Geriatrics, Center for Alzheimer Research, Department of Neurobiology, Care Sciences and Society, Karolinska Institute, Solna, Sweden	Revision of the manuscript for content, including medical writing for content
Johan Skoog	Centre for Ageing and Health (AgeCap), Neuropsychiatric Epidemiology Unit, Department of Psychiatry and Neurochemistry, Institute of Neuroscience and Physiology, Sahlgrenska Academy at the University of Gothenburg, Mölndal; Department of Psychology, University of Gothenburg, Göteborg, Sweden	Revision of the manuscript for content, including medical writing for content
Olof Lindberg	Division of Clinical Geriatrics, Center for Alzheimer Research, Department of Neurobiology, Care Sciences and Society, Karolinska Institute, Solna; Centre for Ageing and Health (AgeCap), Neuropsychiatric Epidemiology Unit, Department of Psychiatry and Neurochemistry, Institute of Neuroscience and Physiology, Sahlgrenska Academy at the University of Gothenburg, Mölndal, Sweden	Revision of the manuscript for content, including medical writing for content
Joana B. Pereira	Neuro Division, Department of Clinical Neurosciences, Karolinska Institute, Stockholm, Sweden	Revision of the manuscript for content, including medical writing for content
Sara Shams	Division of Clinical Geriatrics, Center for Alzheimer Research, Department of Neurobiology, Care Sciences and Society, Karolinska Institute, Solna, Sweden	Revision of the manuscript for content, including medical writing for content
Mana Shams	Division of Clinical Geriatrics, Center for Alzheimer Research, Department of Neurobiology, Care Sciences and Society, Karolinska Institute, Solna, Sweden	Revision of the manuscript for content, including medical writing for content
Miia Kivipelto	Division of Clinical Geriatrics, Center for Alzheimer Research, Department of Neurobiology, Care Sciences and Society, Karolinska Institute, Solna; FINGERS Brain Health Institute, Stockholm; Medical Unit Aging, Karolinska University Hospital, Solna, Sweden; Ageing Epidemiology (AGE) Research Unit, School of Public Health, Imperial College London, Medical School Building, St Mary's Hospital, United Kingdom; Institute of Public Health and Clinical Nutrition and Institute of Clinical Medicine, Neurology, University of Eastern Finland, Kuopio	Revision of the manuscript for content, including medical writing for content
Therese Rydberg Sterner	Centre for Ageing and Health (AgeCap), Neuropsychiatric Epidemiology Unit, Department of Psychiatry and Neurochemistry, Institute of Neuroscience and Physiology, Sahlgrenska Academy at the University of Gothenburg, Mölndal; Aging Research Center, Department of Neurobiology, Care Sciences and Society, Karolinska Institutet and Stockholm University, Stockholm, Sweden	Revision of the manuscript for content, including medical writing for content
Silke Kern	Centre for Ageing and Health (AgeCap), Neuropsychiatric Epidemiology Unit, Department of Psychiatry and Neurochemistry, Institute of Neuroscience and Physiology, Sahlgrenska Academy at the University of Gothenburg, Mölndal, Sweden	Revision of the manuscript for content, including medical writing for content
Anna Zettergren	Centre for Ageing and Health (AgeCap), Neuropsychiatric Epidemiology Unit, Department of Psychiatry and Neurochemistry, Institute of Neuroscience and Physiology, Sahlgrenska Academy at the University of Gothenburg, Mölndal, Sweden	Revision of the manuscript for content, including medical writing for content
Ingmar Skoog	Centre for Ageing and Health (AgeCap), Neuropsychiatric Epidemiology Unit, Department of Psychiatry and Neurochemistry, Institute of Neuroscience and Physiology, Sahlgrenska Academy at the University of Gothenburg; Department of Psychiatry Cognition and Old Age Psychiatry, Sahlgrenska University Hospital, Region Västra Götaland, Mölndal, Sweden	Revision of the manuscript for content, including medical writing for content; major role in the acquisition of data; study concept or design
Eric Westman	Division of Clinical Geriatrics, Center for Alzheimer Research, Department of Neurobiology, Care Sciences and Society, Karolinska Institute, Solna, Sweden	Revision of the manuscript for content, including medical writing for content; study concept or design

## Glossary

AIC: Akaike Information Criteria
BIC: Bayesian Information Criteria
BMI: body mass index
cSVD: cerebral small vessel disease
EPVS: enlarged perivascular space
FA: fractional anisotropy
GM: gray matter
LCA: Latent Class Analysis
MD: mean diffusivity
MET: metabolic equivalent
MMSE: Mini-Mental State Examination
OR: odds ratio
TIV: total intracranial volume
WM: white matter
WML: WM lesion
